# The Association of Childhood Fitness to Proactive and Reactive Action Monitoring

**DOI:** 10.1371/journal.pone.0150691

**Published:** 2016-03-03

**Authors:** Keita Kamijo, Seongryu Bae, Hiroaki Masaki

**Affiliations:** 1 Faculty of Sport Sciences, Waseda University, Tokorozawa, Saitama, Japan; 2 Department of Functioning Activation, National Center for Geriatrics and Gerontology, Obu, Aichi, Japan; University of Rome, ITALY

## Abstract

Several studies have claimed that the positive association between childhood fitness and cognitive control is attributable to differences in the child’s cognitive control strategy, which can involve either proactive or reactive control. The present study tested this hypothesis by manipulating the probability of trial types during a modified flanker task. Preadolescent children performed mostly congruent and mostly incongruent conditions of the flanker task, with post-error task performance and error negativity/error-related negativity (Ne/ERN) being assessed. Results indicated that greater aerobic fitness was related to greater post-error accuracy and larger Ne/ERN amplitudes in the mostly congruent condition. These findings suggest that higher-fit children might be able to transiently upregulate cognitive control by recruiting reactive control in the mostly congruent condition. Further, greater fitness was related to greater modulation of Ne/ERN amplitude between conditions, suggesting that higher-fit children engaged in more proactive control in the mostly incongruent condition. This study supports the hypothesis that greater childhood fitness is associated with a more flexible shift between reactive and proactive modes of cognitive control to adapt to varying task demands.

## Introduction

For over a decade and a half, it has become increasingly clear that regular physical activity can promote brain health and improve cognitive function in adult populations [1–3]. More recently, research has started to examine this relationship in child populations, demonstrating that greater physical activity levels and greater aerobic fitness are positively associated with cognitive functioning (see [4, 5] for reviews). Although most previous studies have employed cross-sectional designs that compared cognitive performance across lower-fit and higher-fit children, recent longitudinal randomized controlled intervention studies have provided evidence of a causal link between physical activity and changes in cognitive function [6–8]. Specifically, these longitudinal studies indicated that a physical activity intervention leading to increases in aerobic fitness improves higher-order cognitive function, also known as cognitive control, in preadolescent children. These findings emphasize the importance of physical activity, not only for prevention of metabolic syndrome but also for cognitive development and brain health. In light of the worldwide epidemic of childhood inactivity [9, 10], the link between childhood fitness and cognitive control should be further clarified.

Cognitive control refers to the ability to coordinate thought and action to execute goal-directed behaviors [11]. It has been well documented that the prefrontal cortex (PFC), which shows protracted maturation [12], plays a critical role in the effective regulation of cognitive control [11]. Although the underlying mechanisms for the beneficial effects of aerobic fitness are still controversial, nonhuman animal models have suggested that aerobic exercise increases nerve growth factors such as brain-derived neurotrophin factor [13], which promotes synaptic plasticity and neurogenesis [14, 15]. It is likely that exercise-induced increases in aerobic fitness are specifically associated with the development of higher-order cognitive function (i.e., cognitive control), which is supported by one of the last brain regions to mature (i.e., the PFC). Stated differently, aerobic fitness may be essential for development of the cognitive control network, including the PFC, during childhood. The present study was designed to provide additional insight into the association between childhood fitness and cognitive development by focusing on cognitive control strategy use, which is linked to PFC activity [16, 17].

Action monitoring has been considered one of the key aspects of cognitive control, since individuals need to monitor and correct their actions during subsequent environmental interaction to optimize goal-directed behaviors. Several neuroelectric studies using error negativity (Ne) [18] or error-related negativity (ERN) [19] have indicated that aerobic fitness is associated with action monitoring system efficiency in preadolescent children [20, 21]. The Ne/ERN is a negative-going component of the response-locked event-related brain potential (ERP) which is elicited following commission errors during a cognitive task. The Ne/ERN has a fronto-central scalp distribution, and is thought to reflect activity of the anterior cingulate cortex (ACC) [22–24], which plays a crucial role in action monitoring [22, 25]. Hillman and his colleagues have indicated that higher-fit children exhibit smaller Ne/ERN amplitude with superior task performance relative to their lower-fit counterparts [20, 21]. These results suggest that greater childhood fitness is associated with less ACC activation, which is thought to reflect more efficient action monitoring (i.e., superior cognitive control).

Several researchers have proposed a possible explanation for the association between childhood fitness and cognitive control [8, 21, 26, 27], which is based on the dual mechanisms of control (DMC) theory [16, 17]. They propose that the association can be attributed to differences in the child’s cognitive control strategy, which can involve either proactive or reactive control. Proactive control is associated with sustained lateral PFC activation, which is linked to decreased transient ACC activation, to anticipate and prevent interference before it occurs. By contrast, reactive control is associated with transient activation of the lateral PFC and a wider brain network including the ACC to detect and resolve interference on an as-needed basis. Based on this theory, smaller Ne/ERN amplitude (i.e., less ACC activation) for higher-fit children is hypothesized to reflect more efficient action monitoring due to their utilization of a proactive control strategy. Using a cue-probe task (i.e., an AX-continuous performance task), Chatham, Frank [28] indicated that young children (3 years) used more probe-driven reactive control, whereas older children (8years) engaged in more cue-driven proactive control. An fMRI study that compared Stroop task related brain activity across adolescents (14–17 years) and young adults (18–25 years) indicated that the neural systems underlying proactive control are still underdeveloped in adolescents [29]. Thus, these developmental studies suggest that proactive control mechanisms show protracted maturation relative to reactive control mechanisms, and that they continue to mature through childhood and into early adulthood. As mentioned earlier, the findings of longitudinal studies [6–8] imply that childhood fitness is associated with development of the cognitive control network involving the PFC. In accordance with these findings, a hypothesized association between childhood fitness and cognitive control strategy use appears to be reasonable.

To test this hypothesis, the present study examined the association between childhood fitness and action monitoring by manipulating the probability of trial types during a modified flanker task. In neuroelectric studies which reported a difference in Ne/ERN amplitude between lower-fit and higher-fit children [20, 21], congruent and incongruent trials were presented equally during the flanker task. An fMRI study [30] manipulated the probability of trial types during the Stroop task and indicated that young adult participants exhibited a smaller interference effect on reaction time (RT; i.e., incongruent RT minus neutral RT) in the mostly incongruent (MI; 70% of trials were incongruent) condition, relative to the mostly congruent (MC; 70% of trials were congruent) condition, with increased sustained lateral PFC activation and decreased transient ACC activation. Such a pattern of results suggests that participants are biased toward adoption of a proactive control strategy in the MI condition. In the MC condition, by contrast, there was decreased sustained activity in the lateral PFC and increased transient activity in the PFC and ACC (De Pisapia & Braver, 2006), which is thought to reflect a greater utilization of reactive control. In line with this, it has been suggested that interference expectancy can influence reliance on proactive versus reactive modes of cognitive control [31]. Specifically, young adult participants should engage in more proactive control when interference is frequent and expected, whereas reactive control should be dominant when interference is infrequent and unexpected. Thus, it is likely that young adults change cognitive control strategy based on the probability of trial types, in order to adapt to varying task demands. If childhood fitness is associated with cognitive development, higher-fit children should show a pattern more similar to that observed in young adults relative to lower-fit children, based on trial type probabilities.

An fMRI study compared brain activity between lower-fit and higher-fit children during the MC and MI conditions of a modified flanker task [26]. This study found greater ACC activity for higher-fit children, relative to lower-fit children, in the MC condition, whereas no such group difference was observed in the MI condition. This pattern of results, coupled with a smaller interference effect on accuracy for higher-fit children, suggests that higher-fit children engaged in more reactive control strategies in the MC condition relative to lower-fit children and flexibly changed cognitive control strategies to adapt to the increased task demands in the MI condition. However, higher-fit children also exhibited an increased sustained PFC activation relative to lower-fit children in the MC condition [26], which contradicts the DMC theory. One possible source of this discrepancy is that this fMRI study used a block design, which could not dissociate transient from sustained brain activation.

The current study further examined the association of childhood fitness to cognitive control strategy using the Ne/ERN, which is thought to be suitable to assess transient brain activation due to its high temporal resolution. We predicted that greater aerobic fitness would be related to smaller Ne/ERN amplitudes in the MI condition, reflecting a greater utilization of a proactive control strategy, whereas such a relationship should be attenuated or even reversed in the MC condition. Additionally, we used post-error accuracy and post-error slowing as behavioral indices of action monitoring. It is well known that individuals show reduced response speed following an error of commission [32], possibly to prevent subsequent errors during cognitive tasks. That is, greater post-error behavioral adjustments are considered to reflect greater utilization of a reactive control strategy. Accordingly, we predicted that greater aerobic fitness would be related to greater post-error behavioral adjustments in the MC condition, whereas such a relationship should be attenuated in the MI condition.

## Methods

### Participants

Fifty preadolescent children participated in this study. The participants also performed an AX-continuous performance task in order to investigate a different cognitive process (i.e., task preparation), and as such, we will report these results elsewhere. Given that weight status has been associated with cognitive control during childhood [33], data from three obese children and one underweight child, as defined by the national cutoff points [34], were excluded from the analyses. Additionally, data from four participants were discarded due to an insufficient number of error trials to compute Ne/ERN averages (< 6 trials) [35, 36]. Thus, analyses were conducted for 42 participants (mean age = 10.5 years, *SD* = 1.1). Table 1 summarizes demographic and fitness information for this sample. Prior to testing, legal guardians reported that their children were free of neurological diseases or physical disabilities and had normal or corrected-to-normal vision. All participants provided written assent and their legal guardians provided written informed consent in accordance with the Ethics Committee on Human Research of Waseda University.

**Table 1:** Mean (*SD*) values for participant demographics and fitness data.

Measure	All participants		Girls		Boys	
No. of participants	42		19		23	
Mean age (years)	10.5	(1.1)	10.2	(0.9)	10.7	(1.1)
20-m shuttle run (#laps)	51.3	(22.0)	42.9	(19.5)	58.2	(21.9)
20-m shuttle run (percentile)	53.3	(32.3)	54.1	(32.9)	52.7	(32.6)
Body mass index (kg/m^2^)	16.8	(1.6)	16.5	(1.2)	17.0	(1.9)
Maternal education	2.8	(0.9)	2.8	(0.8)	2.8	(1.0)
ADHD^a^	7.8	(6.3)	7.1	(6.9)	8.4	(5.8)

^a^Raw scores on the Attention Deficit Hyperactivity Disorder Rating Scale IV.

### Laboratory Procedure

After providing informed consent, participants had their height and weight measured using a Tanita WB-3000 digital scale (Tanita Corp., Tokyo, Japan). Participants’ legal guardians completed the Attention Deficit Hyperactivity Disorder Rating Scale IV [37] and the Physical Activity Readiness Questionnaire [38] to screen for any previous health issues that might be exacerbated by exercise. Additionally, given that socioeconomic status has been associated with cognitive control [39] and fitness [40], maternal educational attainment was assessed as a proxy for socioeconomic status. Maternal education was assessed using a five-point scale ranging from 1, indicating that they did not complete high-school, to 5, indicating an advanced degree. Participants were then fitted with a 64-channel headcap with Ag/AgCl active electrodes (BioSemi ActiveTwo system, Amsterdam, the Netherlands) and seated in a sound-attenuated room where the flanker task took place. Participants were then given instructions, afforded the opportunity to ask questions, and practiced the task prior to the start of testing. The 20-m shuttle run test was conducted on a different day.

### Flanker Task

A modified flanker task consisting of five left- or right-oriented fish was used. This task asked participants to press a button with their index fingers as accurately and quickly as possible corresponding to the direction of a centrally presented target fish and to ignore the flanking fish. The target fish was surrounded by flanking fish that either pointed in the same direction (congruent trials) or the opposite direction (incongruent trials). The flanker task was performed under two conditions in which the probability of congruent and incongruent trials was manipulated. In the MC condition, 70% of trials were congruent and 30% were incongruent, whereas in the MI condition, 30% of trials were congruent and 70% were incongruent. The order of conditions was counterbalanced across the participants. After 40 practice trials, participants completed 320 experimental trials (160 trials × 2 blocks) in each condition. The viewing distance was 1 m and the stimuli subtended a horizontal visual angle between the two outside positions of 9.7° and a vertical visual angle of 1.2°. Stimulus duration was 200 ms, with a randomized stimulus onset asynchrony (SOA) between 1500 and 1900 ms (mean = 1700 ms).

### Task Performance

Post-error accuracy was defined as the percentage of correct responses following trials with errors of commission. Post-error slowing was defined as the mean RT for correct trials following an error of commission trial minus the mean RT for correct trials following a correct trial. Post-error task performance was calculated across congruency due to an insufficient number of errors of commission for the congruent trials.

### ERP Recording

**Fig 1:**
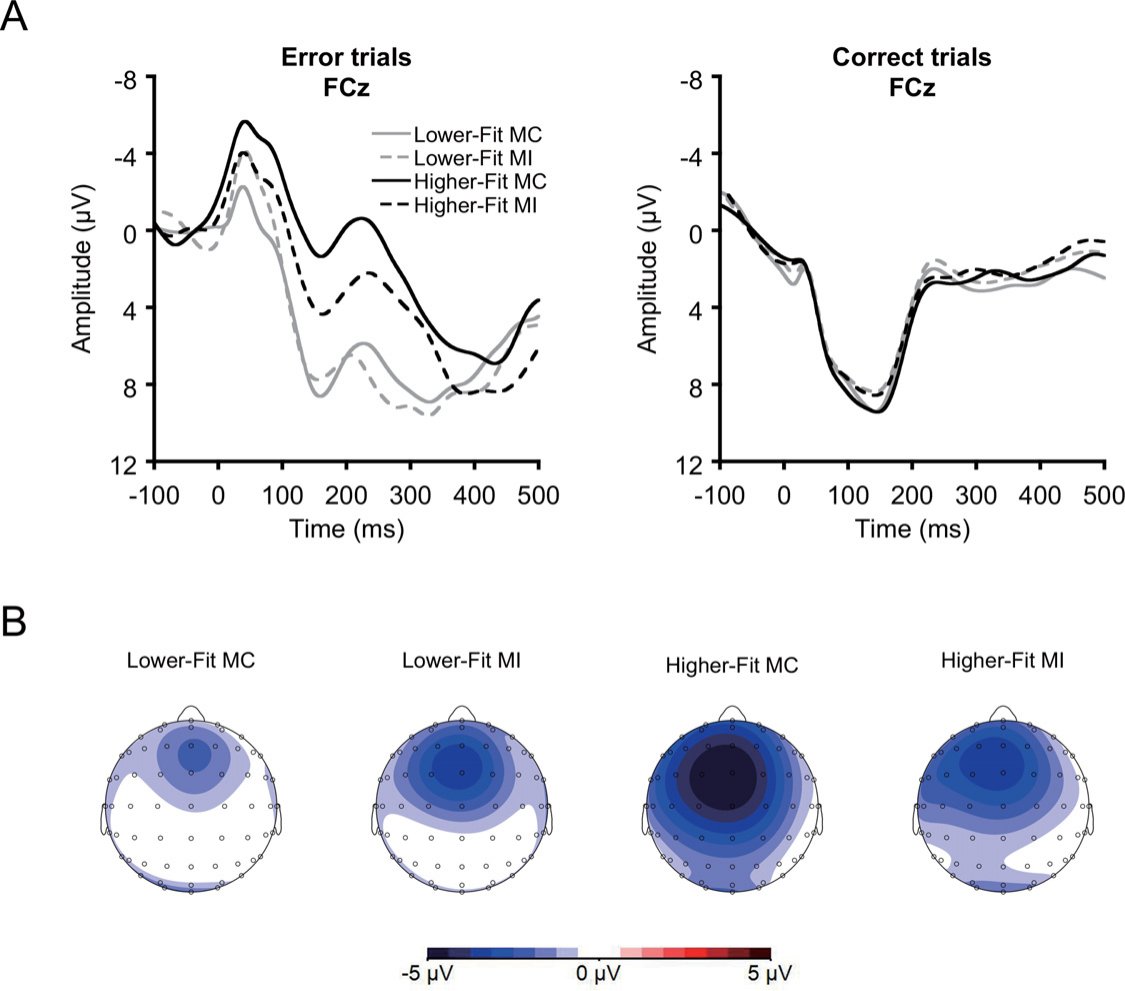
A: Grand averaged response-locked ERP waveforms for error trials (Ne/ERN) and correct trials (Nc/CRN) for each condition at FCz electrode site. B: Topographical maps of the Ne/ERN amplitudes for each condition.

EEG activity was measured from 64 electrode sites arranged in an extended montage based on the International 10–10 system [41], as well as two electrodes placed on the right and left mastoids. Electrooculographic activity was collected from electrodes placed above and below the right orbit and on the outer canthus of each eye to record bipolar eye movements. Continuous data were recorded with a bandwidth of DC to 208 Hz (–3 dB/octave), using the BioSemi Active Two system and sampled at 1024 Hz. Offline EEG processing, which was performed using Brain Vision Analyzer 2 software (Brain Products, Gilching, Germany), included eye movement correction using the procedure described by Gratton, Coles [42], re-referencing to average mastoids, creation of response-locked epochs (–450 to 650 ms relative to response onset), baseline correction (−100 to 0 ms relative to response onset), bandpass filtering (1–12 Hz, 24 dB⁄octave), and artifact rejection (epochs with signals that exceeded ± 100 μV were rejected). Average ERP waveforms were created for error of commission trials (i.e., Ne/ERN) and correct trials (i.e., correct negativity/correct response negativity: Nc/CRN) [43]. Trials with an error of omission were rejected and the waveforms were averaged across congruency. Across conditions, a mean of 31 and 278 trials were averaged for Ne/ERN and Nc/CRN, respectively. Ne/ERN amplitude was assessed at the FCz electrode site, where it reached its topographic maximum (see Fig 1B), and was quantified as the mean voltage within a 20 to 80 ms latency window relative to response onset.

### Fitness Assessment

Participants’ fitness was assessed using the multistage 20-m shuttle run test (also known as the Progressive Aerobic Cardiovascular Endurance Run: PACER). The 20-m shuttle run test was performed according to Leger, Mercier [44]. Participants were required to run back and forth between two lines 20 m apart paced by a tone on a CD player signaling when they should reach the opposite line. The initial speed was set at 8.5 km/h, with the speed increasing by 0.5 km/h every minute. The test was continued until the participant stopped due to fatigue or could not reach the end lines concurrent with the audio signals on two consecutive occasions. The finished lap number was recorded. To exclude age- and sex-related differences, in this study the age- and sex-specific percentile score was calculated as an index of aerobic fitness based on normative data provided by the Japanese Ministry of Education, Culture, Sports, Science and Technology [45].

### Statistical Analysis

Initial Pearson product-moment correlations were computed between demographic factors and dependent variables (i.e., post-error task performance and Ne/ERN amplitude). Hierarchical linear regression analyses were then performed for each dependent variable. Age, sex (dummy coded, 0 = girls, 1 = boys), and maternal education were included in step 1 as control variables, and aerobic fitness (20-m shuttle run test percentile score) was then added to step 2 of the analysis. The significance of the change in the *R*^2^ value between the two steps was used to judge the independent contribution of the fitness measure for explaining variance in the dependent variables. All statistical analyses were conducted using a significance level of *p* = .05.

## Results

### Task Performance

Results of the correlational analysis for post-error task performance and Ne/ERN amplitude are provided in Table 2. The correlation analysis revealed that greater fitness was related to greater post-error accuracy for the MC condition, *r* = .28, *p* = .035 (one-tailed), whereas no such relationship was observed for the MI condition, *r* = .12, *p* = .23. The regression analysis yielded no significant relationship between fitness and post-error accuracy for both MC and MI conditions. The correlation and regression analyses of post-error slowing revealed no significant relationship with fitness for either the MC or MI condition.

**Table 2:** Pearson product-moment correlation coefficients between variables.

Variable		1	2	3	4	5	6	7	8	9	10
1.	Fitness	—									
2.	Age	–.20	—								
3.	Sex^a^	–.02	.26	—							
4.	Maternal education	.10	–.45*	–.04	—						
5.	MC. Post-error accuracy	.28^†^	–.26^†^	–.24	.31*	—					
6.	MI. Post-error accuracy	.12	–.13	–.24	.16	.66*	—				
7.	MC. Post-error slowing	.05	–.16	–.21	.08	.01	–.07	—			
8.	MI. Post-error slowing	.19	.00	–.34*	–.19	.17	.42*	.25	—		
9.	MC. Ne/ERN amplitude	–.43*	–.24	–.19	.24	–.06	.05	.24	–.04	—	
10.	MI. Ne/ERN amplitude	–.18	–.27^†^	–.16	.18	.15	.01	.25	–.16	.64*	—

^a^Sex was dummy coded, 0 = girls, 1 = boys.

*Two-tailed *p* ≤ .05.

^†^One-tailed *p* ≤ .05.

### Ne/ERN

Fig 1A illustrates grand averaged response-locked ERP waveforms for error trials (Ne/ERN) and correct trials (Nc/CRN) for each condition at the FCz electrode site, on which a median split was performed on the 20-m shuttle run test percentile scores within each sex to visualize the association between fitness and Ne/ERN amplitude. Preliminary Bonferroni-corrected *t*-tests were conducted within each condition comparing Ne/ERN amplitude with Nc/CRN amplitude. Results indicated larger Ne/ERN amplitude relative to Nc/CRN amplitude across conditions, *t*s(42) ≥ 7.0, *p*s ≤ .001, confirming the expected accuracy effect.

A median split was performed on the 20-m shuttle run test percentile scores within each sex to visualize the association between fitness and Ne/ERN amplitude.

The correlation analysis revealed that greater fitness was related to larger Ne/ERN amplitude for the MC condition, *r* = –.43, *p* = .004, whereas no such relationship was observed for the MI condition, *r* = –.18, *p* = .27 (Table 2). A summary of the regression analyses for Ne/ERN amplitude for each condition is provided in Table 3. The regression analysis for the MC condition yielded a significant change in *R*^2^ at step 2, *F*(1, 37) = 13.3, *p* = .001, indicating that greater fitness was associated with larger Ne/ERN amplitude. For the MI condition, no significant fitness influence was present at step 2, *F*(1, 37) = 2.3, *p* = .14. An additional multiple hierarchical regression analysis was conducted to examine the difference in Ne/ERN amplitude between the MC and MI conditions (i.e., MC–MI). This analysis tested for a possible relationship between fitness and differences in ACC activity, which is thought to reflect the flexibility to change cognitive control strategy use based on the probability of trial types. This analysis yielded a significant change in the *R*^2^ at step 2, *F*(1, 37) = 4.0, *p* = .05, indicating that greater fitness was associated with greater modulation of Ne/ERN amplitude between the MC and MI conditions.

**Table 3:** Summary of regression analyses for variables predicting Ne/ERN amplitude.

		MC		MI		MC–MI^a^	
		Δ*R*^2^	β	Δ*R*^2^	β	Δ*R*^2^	β
Step1		.10		.09		.02	
	Age		–.12		–.21		.11
	Sex		–.16		–.10		–.07
	Maternal education		.18		.08		.13
Step 2		.24*		.05		.10*	
	Fitness		–.50*		–.24		–.32*

^a^A difference in Ne/ERN amplitude between the MC and MI conditions.

*p ≤ .05.

## Discussion

The main findings here were that greater aerobic fitness was related to larger Ne/ERN amplitude, which reflects increased transient ACC activation, in the MC condition. Additionally, although the regression analysis revealed no relationship between fitness and post-error task performance, correlation analyses implied that greater aerobic fitness was associated with greater post-error accuracy in the MC condition. Based on the DMC theory [16, 17, 30], these findings suggest that higher-fit children might be able to maintain greater post-error accuracy in the MC condition relative to lower-fit children by recruiting reactive control, as denoted by larger Ne/ERN amplitude. This interpretation can also be accounted for by conflict monitoring theory [46]. This theory proposes that the ACC monitors response conflict on error trials and provides a signal to the dorsolateral PFC to upregulate cognitive control in support of behavioral adjustments on subsequent trials [25, 46]. Accordingly, we suggest that the observed larger Ne/ERN amplitude and greater post-error accuracy in the MC condition for higher-fit children represent transient intensification of cognitive control due to greater utilization of reactive control.

More importantly, greater fitness was associated with greater modulation of Ne/ERN amplitude between the MC and MI conditions. Stated differently, higher-fit children had smaller Ne/ERN amplitude in the MI condition relative to the MC condition, whereas such a relationship was attenuated for lower-fit children. Based on the DMC theory [16, 17, 30], it is plausible that the decreased Ne/ERN amplitude (i.e., less ACC activation) in the MI condition for higher-fit children is due to increased sustained PFC activation, reflecting a greater utilization of proactive control. Thus, these findings suggest that greater childhood fitness is associated with a more flexible shift between reactive and proactive modes of cognitive control based on the probability of trial types.

However, it is noteworthy that, contrary to our hypothesis, aerobic fitness was not related to Ne/ERN amplitude in the MI condition. Pontifex, Raine [21] manipulated stimulus-response compatibility during a modified flanker task, and observed smaller Ne/ERN amplitude and superior task performance, which may reflect recruitment of proactive control by higher-fit children, relative to lower-fit children, in the stimulus-response compatible condition. Higher-fit children also exhibited superior task performance in the stimulus-response incompatible condition, but had increased Ne/ERN amplitude that did not differ from lower-fit children. These findings imply that higher-fit children engaged more in reactive control in the more effortful condition (i.e., incompatible condition) to meet the increased task demands. Although it remains unclear whether proactive control and reactive control are fully independent, they may be able to be engaged simultaneously [16, 17]. Taken together, in the present study, we speculate that higher-fit children might engage in both proactive and reactive control simultaneously in the more demanding condition (i.e., MI condition), and therefore aerobic fitness was not related to Ne/ERN amplitude. Given that this is merely speculation, further studies are required to confirm this interpretation using fMRI techniques to assess both transient and sustained brain activation.

Given the cross-sectional design used here, this study does not demonstrate a causal relationship between changes in childhood fitness and cognitive control strategy use. A randomized controlled intervention study [8] showed that a physical activity program, one that lead to increases in aerobic fitness, improved working memory performance on a modified Sternberg task. Further, the program improved the effectiveness of task preparation, as assessed by contingent negative variation (CNV). The CNV is a negative slow potential that develops during the foreperiod between warning and imperative stimuli, and is composed of at least two different components, the early and late CNVs [47, 48]. Kamijo, Pontifex [8] indicated that the physical activity program selectively enhanced early but not late CNV amplitude. An fMRI study manipulated participants’ cognitive control strategy (i.e., proactive or reactive) during the Sternberg task, and observed increased sustained PFC activation during the foreperiod with superior task performance when participants engaged in proactive control, relative to reactive control [49]. Based on this study, it is plausible that the selective effect of the physical activity program on the early CNV, which was accompanied by improvement in task performance, indicates a strategy shift from reactive control to proactive control due to increased fitness. Thus, although this longitudinal study did not manipulate cognitive control strategies [8], these findings imply a causal relationship between fitness and cognitive control strategy use during childhood.

A cross-sectional study has supported the association between childhood fitness and cognitive control strategy by focusing on the effectiveness of task preparation [27]. Berchicci, Pontifex [27] assessed the Bereitschaftspotential (BP) and the prefrontal negativity (pN) during a modified flanker task. The BP and pN are negative slow potentials that develop around 1.5 to 1 s before movement onset, and believed to reflect responses [50] and cognitive preparation [27, 51], respectively. Berchicci, Pontifex [27] indicated that higher-fit children exhibited larger pN relative to their lower-fit counterparts, whereas no such difference was observed for the BP. The larger pN observed in higher-fit children might reflect more effective cognitive preparation by their utilization of a proactive control strategy, which is consistent with the above described CNV study [8]. Note that although we also assessed the BP and pN in the present study, no association between fitness and pN/BP amplitude was observed (S1 Text). The implication of this discrepancy might be related to the difference in SOA used for the flanker task. The SOA was randomized in the present study, whereas it was fixed in the previous study [27]. A randomized SOA might make it more difficult to prepare for upcoming stimuli. We believe that in the present study fitness was not associated with cognitive preparation processes (i.e., pN amplitude) for this reason.

As stated in the introduction, animal studies suggest that exercise induced increases in nerve growth factors relate to increases in the number of synaptic connections and the development of new neurons that support learning and memory [14, 15]. If these changes were to occur in the human brain, it is no wonder that cognitive control strategies are susceptible to the effect of aerobic fitness, given that the neural networks underlying cognitive control strategies are still underdeveloped during childhood [29]. It is reasonable to suggest that the present results for neuroelectric (i.e., Ne/ERN) and behavioral (i.e., post-error accuracy) measures of action monitoring indicate differences in children’s cognitive control strategies based on their fitness level. Nonetheless, additional longitudinal studies are needed to examine how changes in physical activity and fitness influence cognitive control strategies during childhood.

Another limitation is that findings of previous neuroelectric studies [20, 21] that observed smaller Ne/ERN amplitudes for higher-fit relative to lower-fit children would appear to be inconsistent with the present results. In the previous studies’ flanker task, congruent and incongruent trials were presented equally often. Given that we did not employ a 50/50 condition in this present study and did find that the association between childhood fitness and action monitoring differed based on the probability of trial types, it is difficult to compare and contrast study outcomes among these neuroelectric studies.

## Conclusion

On the basis of results from neuroelectric and behavioral measures of action monitoring, we suggest that higher-fit children showed a strategic shift from use of reactive control in the MC condition to increasing use of proactive control in the MI condition. The shift in the cognitive control mode based on the probability of trial types for higher-fit children is similar to that observed in young adults in a previous study [30]. Thus, the current findings suggest that childhood fitness may be associated with development of the neural network underlying cognitive control strategies. In conclusion, this study suggests that greater childhood fitness is associated with a more flexible shift between reactive and proactive modes of cognitive control to adapt to varying task demands.

## Supporting Information

S1 Dataset Demographic, fitness, behavioral, and neuroelectric data for the 42 participants. (XLSX)

S1 Fig A: Grand averaged stimulus-locked ERP waveforms for each condition at Fpz (pN) and Cz (BP) electrode site. B: Topographical maps of the pN/BP amplitudes for each condition.
(PDF)

S1 Text pN/BP analysis and results. (DOCX)
