# Supplementary material for: The Association of Childhood Fitness to Proactive and Reactive Action Monitoring
Source: PLoS One. 2016 Mar 3;11(3):e0150691. doi: 10.1371/journal.pone.0150691 (PMC4777555; doi:10.1371/journal.pone.0150691)
Supplement: S1 Fig — A: Grand averaged stimulus-locked ERP waveforms for each condition at Fpz (pN) and Cz (BP) electrode site. B: Topographical maps of the pN/BP amplitudes for each condition. (PDF) [file pone.0150691.s002.pdf]

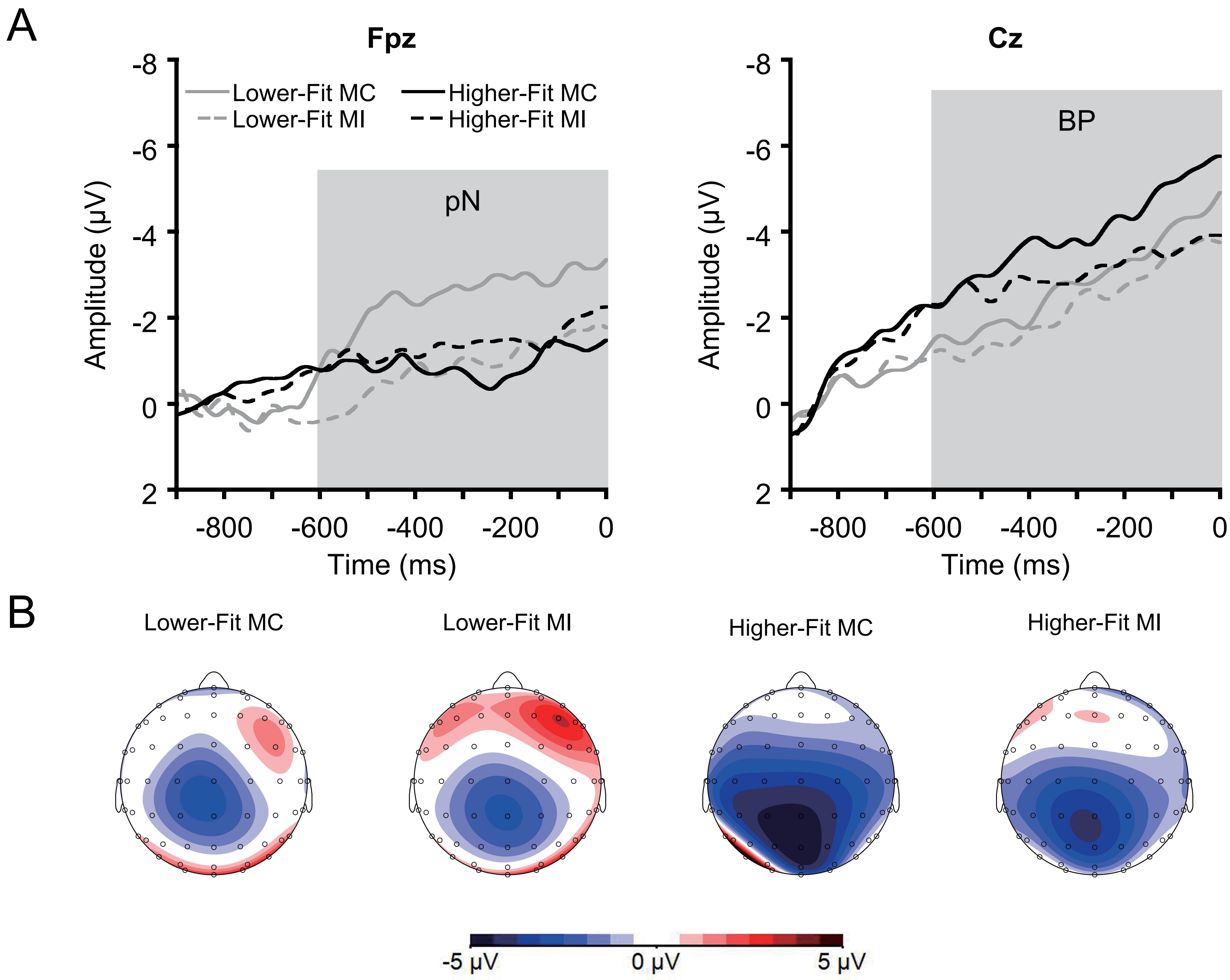


**S1 Figure. A: Grand averaged stimulus-locked ERP waveforms for each condition at Fpz (pN) and Cz (BP) electrode site. B: Topographical maps of the pN/BP amplitudes for each condition.**

A median split was performed on the 20-m shuttle run test percentile scores within each sex to visualize the association between fitness and pN/BP amplitude.
