# Supplementary material for: The Association of Childhood Fitness to Proactive and Reactive Action Monitoring
Source: PLoS One. 2016 Mar 3;11(3):e0150691. doi: 10.1371/journal.pone.0150691 (PMC4777555; doi:10.1371/journal.pone.0150691)
Supplement: S1 Text — (DOCX) [file pone.0150691.s003.docx]

**pN/BP Analysis**

Offline EEG processing, which was performed using Brain Vision Analyzer 2 software (Brain Products, Gilching, Germany), included eye movement correction using the procedure described by Gratton, Coles [42], re-referencing to average mastoids, creation of stimulus-locked epochs (–900 to 900 ms relative to stimulus onset), baseline correction (−900 to −800 ms relative to stimulus onset), low-pass filtering (10 Hz, 24 dB⁄octave), and artifact rejection (epochs with signals that exceeded ± 100 µV were rejected). Trials with a response error were rejected and the waveforms were averaged across congruency. Data from one participants were discarded due to excessive noise in the EEG signal. Thus, pN/BP analyses were conducted for 41 participants. Across conditions, a mean of 223 trials was averaged for pN/BP. Based on Berchicci, Pontifex [27], pN and BP were assessed at the Fpz and Cz respectively, and were quantified as the mean voltage within −600 to 0 ms latency window relative to stimulus onset. We used the same statistical approach for pN/BP amplitude as used for Ne/ERN amplitude.

**pN/BP Results**

S1 Figure illustrates grand averaged stimulus-locked ERP waveforms for each condition at the Fpz and Cz electrode sites, on which a median split was performed on the 20-m shuttle run test percentile scores within each sex to visualize the association between fitness and pN/BP amplitude. The correlation and regression analyses of pN and BP amplitude revealed no significant relationship with fitness for either the MC or MI condition (Table A and B).

**Table A. Pearson product-moment correlation coefficients between variables.**

| Variable | | 1 |  | 2 |  | 3 |  | 4 |  | 5 |  | 6 |  | 7 |  | 8 |  |
| --- | --- | --- | --- | --- | --- | --- | --- | --- | --- | --- | --- | --- | --- | --- | --- | --- | --- |
| 1. | Fitness | — | |  |  |  |  |  |  |  |  |  |  |  |  |  |  |
| 2. | Age | –.16 |  | — | |  |  |  |  |  |  |  |  |  |  |  |  |
| 3. | Sex^a^ | .01 |  | .23 |  | — | |  |  |  |  |  |  |  |  |  |  |
| 4. | Maternal education | .07 |  | –.43 | ^*^ | –.01 |  | — | |  |  |  |  |  |  |  |  |
| 5. | MC. pN amplitude | .17 |  | –.11 |  | –.15 |  | –.07 |  | — | |  |  |  |  |  |  |
| 6. | MI. pN amplitude | .01 |  | .00 |  | –.09 |  | .03 |  | .50 | ^*^ | — | |  |  |  |  |
| 7. | MC. BP amplitude | –.22 |  | .21 |  | .16 |  | –.01 |  | –.01 |  | .29 | ^†^ | — | |  |  |
| 8. | MI. BP amplitude | –.04 |  | .18 |  | .24 |  | –.04 |  | .02 |  | .38 | ^*^ | .64 | ^*^ | — | |

^a^Sex was dummy coded, 0 = girls, 1 = boys.

^*^Two-tailed *p* < .05.

^†^One-tailed *p* < .05.**Table B. Summary of regression analyses for variables predicting pN and BP amplitude.**

|  |  | pN_MC | |  | pN_MI | |  | BP_MC | |  | BP_MI | |
| --- | --- | --- | --- | --- | --- | --- | --- | --- | --- | --- | --- | --- |
|  |  | ∆*R*^2^ | β |  | ∆*R*^2^ | β |  | ∆*R*^2^ | β |  | ∆*R*^2^ | β |
| Step1 | | .04 |  |  | .01 |  |  | .06 |  |  | .07 |  |
|  | Age |  | –.14 |  |  | .04 |  |  | .22 |  |  | .14 |
|  | Sex |  | –.12 |  |  | –.10 |  |  | .11 |  |  | .21 |
|  | Maternal education |  | –.13 |  |  | .05 |  |  | .09 |  |  | .02 |
| Step 2 | | .03 |  |  | .00 |  |  | .04 |  |  | .00 |  |
|  | Fitness |  | .16 |  |  | .01 |  |  | –.20 |  |  | –.02 |
